# Outcomes for Dual-Eligible Beneficiaries With Dementia in Special Needs Plans and Other Medicare Advantage Plans

**DOI:** 10.1001/jamanetworkopen.2024.61219

**Published:** 2025-02-21

**Authors:** Wendy Xu, Eli Raver, Caroline Carlin, Roger Feldman, Sheldon M. Retchin, Jeah Jung

**Affiliations:** 1 Division of Health Services Management and Policy, College of Public Health, The Ohio State University, Columbus; 2 Department of Family Medicine and Community Health, School of Medicine, University of Minnesota, Twin Cities; 3 Division of Health Policy and Management, School of Public Health, University of Minnesota, Twin Cities; 4 Division of General Internal Medicine, College of Medicine, The Ohio State University, Columbus; 5 Department of Health Administration and Policy, College of Public Health, George Mason University, Fairfax, Virginia

## Abstract

**Importance:**

Alzheimer disease and related dementias (ADRD) are especially prevalent among Medicare-Medicaid dual-eligible beneficiaries—the clinical complexity of ADRD amplifies the challenges of managing chronic conditions and accessing care for dual-eligible beneficiaries, and the need to navigate the fragmented Medicare-Medicaid benefits adds to patients’ burdens. Little is known about how enrollment in dual-eligible special needs plans (D-SNPs) that coordinate Medicare and Medicaid coverage is associated with health outcomes for dual-eligible beneficiaries with ADRD.

**Objective:**

To examine the associations between Medicare Advantage (MA) plan type and adverse medical events among dual-eligible beneficiaries with ADRD.

**Design, Setting, and Participants:**

This cross-sectional analysis used MA encounter data from 2016 to 2019. Participants included dual-eligible, community-dwelling Medicare beneficiaries aged 65 years and older with ADRD who enrolled in MA plans in 50 US states and Washington, DC. Data were analyzed from January to November 2024.

**Exposures:**

Enrollment in non–D-SNP Medicare Advantage plans and D-SNPs. The exposure variable for additional analyses included enrollment in fully integrated special needs plans (FIDE SNPs).

**Main Outcomes and Measures:**

Preventable hospitalizations, 30-day all-cause readmissions, and avoidable emergency department (ED) visits.

**Results:**

Analysis included a total of 121 145 patients in non–D-SNP plans with an enrollment of 173 662 patient-years (mean [SD] age, 81.8 [7.8] years; 122 565 female [70.6%]; 6078 Asian [3.5%], 34 150 Black [19.7%], 37 580 Hispanic [21.6%], 95 854 White [55.2%]) and 78 166 patients in D-SNP plans with an enrollment of 122 681 patient-years (mean [SD] age, 80.2 [8.0] years; 87 329 female [71.2%]; 10 530 Asian [8.6%], 33 280 Black [27.1%], 43 294 Hispanic [35.3%], 35 577 White [29.0%]). Dual-eligible enrollees with ADRD had a preventable hospitalization rate of 10.8% (95% CI, 10.3%-11.4%), a 30-day readmission rate of 22.3% (95% CI, 21.6%-22.9%), and an avoidable ED visit rate of 21.1% (95% CI, 20.3-21.9) in D-SNPs. These rates were 11.1% (95% CI, 10.7%-11.5%), 23.6% (95% CI, 22.9%-24.3%), and 20.8% (95% CI, 20.1%-21.6%) in non–D-SNP MA plans. There were no statistical differences in rates of preventable hospitalizations or avoidable ED visits between dual-eligible enrollees with ADRD in D-SNPs and those in non–D-SNP MA plans. Compared with non–D-SNPs, enrollees in FIDE SNPs had lower probabilities of preventable hospitalizations by 1.2 percentage points (95% CI, −2.7 to −0.2 percentage points) and 30-day readmissions by 7.2 percentage points (95% CI, −9.3 to −5.1 percentage points).

**Conclusions and Relevance:**

Dual-eligible beneficiaries with ADRD enrolled in D-SNPs and non–D-SNP MA plans had similar rates of adverse events. Dual-eligible beneficiaries with ADRD may benefit from enrollment in more coordinated MA plans, such as FIDE SNPs, by lowering unnecessary hospitalizations.

## Key Points

**Question:**

What are the associations between Medicare Advantage (MA) plan type and adverse medical events among dual-eligible beneficiaries with Alzheimer disease and related dementias (ADRD)?

**Findings:**

In this cross-sectional analysis, adverse outcomes for dual-eligible beneficiaries with ADRD who enrolled in dual-eligible special needs plans (D-SNPs) did not differ from those in non–D-SNP MA plans. Enrollment in fully integrated SNP plans was associated with fewer preventable hospitalizations and readmissions.

**Meaning:**

These results suggest that dual-eligible beneficiaries with ADRD can benefit from enrollment in more coordinated MA plans by lowering unnecessary hospitalizations.

## Teaser

This cross-sectional analysis of US adults with Alzheimer disease and related dementias dually enrolled in Medicare and Medicaid examines the association of type of Medicare Advantage plan with adverse health outcomes.

## Introduction

Almost 13 million individuals are dually eligible for Medicare and Medicaid. Dually eligible individuals have much worse health than other Medicare beneficiaries and account for a large proportion of total expenditures for the Medicare and Medicaid programs.^1^ Coordinating care for dually eligible beneficiaries is difficult—they are much more likely to rely on emergency departments (EDs) for routine care and are more likely to be admitted to hospitals, often for ambulatory care–sensitive conditions.^2,3,4^ Thus, following the Medicare Modernization Act of 2003, dual-eligible beneficiaries can enroll in a special needs plan (D-SNP), a type of Medicare Advantage (MA) plan designed to better serve their needs. D-SNPs are required to hold contracts with state Medicaid agencies, and they exclusively enroll dual-eligible beneficiaries.

Fifty-one percent of dual-eligible beneficiaries are enrolled in Medicare Advantage plans in 2024.^5^ Dual-eligible beneficiaries can enroll in MA plans that are not D-SNPs (hereafter “non–D-SNP MA plans”). States provide Medicaid coverage that pays for Medicare cost-sharing requirements and certain benefits not included in Medicare. Non–D-SNP MA plans do not attempt to coordinate between Medicare and Medicaid benefits and are not exclusively for dual-eligible beneficiaries. Dual-eligible beneficiaries can also choose D-SNPs, a type of MA plan that covered 1 in 3 dual-eligible beneficiaries in 2021.^5^ D-SNPs and non–D-SNP MA plans operate under the same reimbursement mechanism, receiving risk-adjusted, per-capita payments and bonus payments from Medicare based on quality metrics.^6^ In addition, D-SNPs must include a formal Model of Care approved by the National Committee on Quality Assurance to address the care needs of individual dual-eligible members.^7^ D-SNPs are required to coordinate Medicare and Medicaid benefits, such as helping beneficiaries navigate provider networks, assisting with prior authorization for care access, and coordinating Medicaid’s share of Medicare cost sharing. Enrollment of dual-eligible beneficiaries in D-SNPs is growing rapidly.^5^ While the Centers for Medicare and Medicaid Services (CMS) is actively promoting better integration through D-SNPs, recent studies revealed that dual-eligible beneficiaries in D-SNPs did not have substantially better access to ambulatory care than those not in D-SNPs.^8,9,10^

Among the chronic conditions that affect dual-eligible beneficiaries, Alzheimer disease and related dementias (ADRD) are especially prevalent.^11^ The clinical complexity and irreversible functional dependency of patients with ADRD further amplify the challenges of managing chronic conditions and accessing regular ambulatory care services faced by dual-eligible beneficiaries. The need to navigate the fragmented coverage of Medicare and Medicaid adds to patients’ and caregivers’ burdens for dual-eligible beneficiaries with ADRD. ADRD affected 6 million people in America in 2023,^12^ with an expected increase to 14 million by 2060.^13^ ADRD patients have different and more intensive care patterns, making it challenging to generalize the care experiences of all Medicare beneficiaries to those with ADRD.^14^ Medicare beneficiaries with ADRD were less likely to remain enrolled in MA plans than those without ADRD,^15^ raising concerns of unmet health care needs in MA. Yet most research on adverse medical events for Medicare patients with ADRD is limited to traditional Medicare beneficiaries.^16,17^ Little is known about the adverse outcomes in dual-eligible enrollees with ADRD in D-SNPs compared with those in other MA plans. Given the care complexity and administrative burdens to patients, it is critical to understand how the more coordinated MA plans are associated with care quality for dual-eligible beneficiaries with ADRD.

The objective of this study was to examine the associations between D-SNP and non–D-SNP MA plans and adverse medical events among dual-eligible beneficiaries with ADRD. We hypothesized that patients in D-SNPs were less likely to have avoidable ED visits, preventable hospitalizations, or 30-day readmissions than those in non–D-SNP MA plans. Dual-eligible beneficiaries with ADRD who are frailer and have more demanding health conditions may benefit from enrolling in plans with enhanced coordination of care and benefits. Therefore, we hypothesized that the associations between adverse events and MA plan type varied by patients’ health risks.

Most dual-eligible enrollees are in coordination-only D-SNPs that offer limited integration with Medicaid. A small proportion of beneficiaries are enrolled in fully integrated dual-eligible special needs plans (FIDE SNPs), where a plan’s parent organization must offer a Medicaid plan that covers primary and acute care services as well as long-term services and supports. By design, FIDE SNPs should integrate Medicaid and Medicare services and therefore offer the greatest degree of coordination between Medicare and Medicaid among all D-SNPs. To inform policymakers about the potential effectiveness of FIDE SNPs on outcomes of dual-eligible beneficiaries with ADRD, we conducted an additional analysis that compared non–D-SNP MA plans, coordination-only D-SNPs, and FIDE SNPs.

## Methods

For this cross-sectional study, we used 100% MA encounter data from 2016 to 2019, the latest data available before the COVID-19 pandemic. The 2016 data were only used to identify the ADRD population. We used Master Beneficiary Summary Files for information about beneficiary age, sex, race, zip codes of residence, and monthly Medicare and Medicaid enrollment details. The encounter data captured diagnoses, clinical services, service locations, and dates.

### Study Population

The study population comprised dual-eligible, community-dwelling Medicare beneficiaries aged 65 years and older with ADRD. Medicare beneficiaries who enrolled in either full or partial Medicaid programs any month of a year were considered dual-eligible beneficiaries. Following the algorithm CMS uses for the Medicare population, patients with ADRD were identified by having at least 1 inpatient claim or 2 outpatient claims with ADRD diagnosis codes in a 2-year reference window.^18^ The inclusion criteria required beneficiaries to reside in 50 states or Washington, DC, with continuous Medicare Part A, B, or D coverage. We included only enrollees in MA contracts with highly complete encounter data, following a validated method in prior research.^19,20^ We excluded enrollees who switched between D-SNPs and non–D-SNP MA plans during a year, patients with end-stage kidney disease, and enrollees of Program of All-Inclusive Care for the Elderly plans, cost plans, and private fee-for-service plans. We also excluded enrollees in SNPs designed mainly for institutional care and for those with chronic health conditions (3.4% of the study population). Because D-SNPs are tailored to support care infrastructure for community dwellers,^21^ long-stay nursing home residents were excluded from the study. In the main analysis, we excluded enrollees in FIDE SNPs, which were available in 10 states during our study period, but represented only 4.9% of the identified MA population.^22^ Therefore, D-SNP enrollees in the main analyses were limited to those in coordination-only D-SNPs.^5^ More details about the study population are in eAppendix 1 in Supplement 1. The Ohio State University institutional review board exempted this study from review and informed consent was not needed because we only conducted secondary data analysis. This study followed the Strengthening the Reporting of Observational Studies in Epidemiology (STROBE) reporting guidelines.

### Outcomes

We identified 3 types of adverse medical events that could be avoided with adequate care management and effective, timely ambulatory care: avoidable ED visits, preventable hospitalizations, and 30-day all-cause readmissions. Avoidable ED visits were constructed following a validated and updated algorithm widely used in prior studies.^23,24^ Preventable hospitalizations were identified by the algorithm from the Agency for Healthcare Research and Quality Prevention Quality Indicators.^25^ The algorithm from the CMS hospital readmissions reduction program was used to identify all-cause readmissions within 30 days of discharge.^26^

### Key Explanatory Variable

MA plan type was measured by enrollment in non–D-SNP MA plans and D-SNPs. The additional analysis also included enrollees in FIDE SNPs.

### Covariates

Based on the behavioral model of health services use,^27^ covariates included beneficiary age (continuous variable), sex (female, male), and race and ethnicity defined by the CMS (Hispanic, non-Hispanic Asian or Pacific Islander, non-Hispanic Black, and non-Hispanic White). Following the Medicare algorithm that uses inpatient and outpatient claims and encounter data, beneficiary health risk was measured by the CMS Hierarchical Condition Category (HCC) score, which captures health care resource intensity due to health conditions. We excluded diagnoses listed in medical record reviews or health risk assessment records from computing HCC scores because use of those records substantially varied across MA plans.^28,29,30^ In a sensitivity analysis, we included diagnoses from all data sources, and the findings were consistent with the main results. We also included Kim’s validated claims-based frailty index score for older adults, ranging from 0 to 1.^31^ Other covariates included median zip code income and education levels, rurality, county health care resources including hospital beds and physicians per 1000 population, and the MA county-level Herfindahl-Hirschman Index (HHI).

### Statistical Analysis

We estimated linear probability models for each adverse outcome as a function of D-SNP enrollment, using non–D-SNP MA plans as the comparison. The unit of analysis for avoidable ED visits and preventable hospitalizations was a person-year. The 30-day readmission outcome was analyzed at the hospital admission level per year. In addition to covariates, given the differing intensities of care by geography, we further controlled for county fixed effects. We also controlled for year fixed effects in all estimations. Robust standard errors were clustered by insurance plans.

We used inverse probability of treatment weighting (IPTW) to balance observed characteristics between dual-eligible enrollees with ADRD with different coverage types. Weights were calculated as the inverse of the projected probability of the observed enrollment group based on logistic regression models. Covariate balance before and after weighting was assessed using the standardized mean difference.^32^ IPTW effectively improved the comparability of the enrollment groups, minimizing biases from differences in cohort characteristics between plan types (eTable 1, eFigures 1 and 2 in Supplement 1). The propensity score distributions for the non–D-SNP MA and D-SNP groups showed that there were valid comparisons between groups over a wide range of probabilities of being enrolled in one group or another. Love plots of covariate balance before and after IPTW are also included in eFigure 2 in Supplement 1. These analyses were performed using Stata version 18 (StataCorp). Significance was determined through 95% CIs.

#### Stratified Analyses by Health Risk

We conducted stratified analyses to examine whether the associations between plan type and adverse events varied by enrollees’ projected health risks. For each outcome measure, health risk was categorized as being in the top, middle, or bottom tercile of projected risks of incurring an adverse event, estimated by a logistic regression on the HCC and frailty index scores. The health risk model was developed using a 10% training sample, and projected risks were calculated for the remaining 90% testing sample. Within each projected health risk tercile, we conducted regression analyses to examine associations between plan type and each adverse outcome, controlling for the same set of covariates and county and year fixed effects as in the main analyses, except for HCC and frailty index scores. The sample for each health risk tercile was reweighted using IPTW (multinomial logistic) to balance patient characteristics across plan types within the health risk tercile.

#### Additional Analyses and Sensitivity Analyses

We compared outcomes of FIDE SNP enrollees and those in D-SNPs, respectively, with outcomes of enrollees in non–D-SNP MA plans. This analysis used data from enrollees in 10 states where FIDE SNPs operated during the study period and IPTW was used again.

We also conducted several sensitivity analyses. First, we limited the population to dual-eligible beneficiaries who received full Medicaid benefits, comprising 82% of the study population.^33^ Second, we modified the avoidable ED measure to include ED visits for mental health conditions. This is because behavioral disturbances are one of the major reasons for ED visits among patients with ADRD,^14^ yet behavioral condition-related ED use is not explicitly included in the algorithm we used for the primary avoidable ED use measure.^14^ We also estimated the model using all ED visits. Third, we repeated the analyses using probit models.

## Results

A total of 121 145 patients were in non–D-SNP MA plans with a combined 173 662 patient-years (mean [SD] age, 81.8 [7.8] years; 122 565 female [70.6%]; 6078 Asian [3.5%], 34 150 Black [19.7%], 37 580 Hispanic [21.6%], 95 854 White [55.2%]), and 78 166 patients were in D-SNPs with a combined 122 681 patient-years (mean [SD] age, 80.2 [8.0] years; 87 329 female [71.2%]; 10 530 Asian [8.6%], 33 280 Black [27.1%], 43 294 Hispanic [35.3%], 35 577 White [29.0%]) (Table 1). The 2 plan types had similar population ages, distributions of sex, mean HCC risk scores, and frailty scores. Zip code levels of household income, education, hospital bed density, and physician concentration were all similar across the plan types. The proportion of rural residents was 14 990 of 122 681 patients (12.2%) in D-SNPs and 25 481 of 173 662 patients (14.7%) in non–D-SNP MA plans. Study populations at the admission level for 30-day readmissions are reported in eAppendix 3 in Supplement 1.

**Table 1.** Characteristics of Dually Eligible Patients With ADRD, by Coverage Type, 2017-2019

Characteristics	Non–D-SNP Medicare Advantage plans	D-SNP
Patient-year, No.	173 662	122 681
Patients, No.	121 145	78 166
Health plans, No.	2085	300
Plan-years, No.	3838	575
Age, mean (SD), y	81.8 (7.8)	80.2 (8.0)
Sex, No. (%)		
Female	122 565 (70.6)	87 329 (71.2)
Male	51 097 (29.4)	35 352 (28.8)
Race and ethnicity, No. (%)		
White (non-Hispanic)	95 854 (55.2)	35 577 (29.0)
Black (non-Hispanic)	34 150 (19.7)	33 280 (27.1)
Asian (non-Hispanic)	6078 (3.5)	10 530 (8.6)
Hispanic (any race)	37 580 (21.6)	43 294 (35.3)
Rural, No. (%)	25 481 (14.7)	14 990 (12.2)
HCC score, mean (SD)	2.1 (1.5)	2.2 (1.5)
Frailty score, mean (SD)	0.22 (0.09)	0.22 (0.09)
Median household income (zip code level), mean (SD), $	58 310 (22 703)	50 967 (20 190)
Zip code households with a 4-y degree, mean (SD), %	26.9 (14.4)	24.5 (14.0)
Zip code households speaking only English, mean (SD), %	73.3 (26.0)	67.1 (27.9)
Hospital beds per 1000 population (county level), mean (SD)	3.1 (1.9)	3.4 (1.9)
Physicians per 1000 population (county level), mean (SD)^a^	3.6 (2.2)	3.9 (2.8)
Medicare Advantage county HHI, mean (SD)	2130 (1055)	2005 (1006)

Abbreviations: ADRD, Alzheimer disease and related dementia; D-SNP, dual-eligible special need plans; HCC, Centers for Medicare & Medicaid Services Hierarchical Condition Category; HHI, Herfindahl-Hirschman Index.

^a^ Physicians per 1000 population is the total number of physicians in a county per 1000 county population.

Dual-eligible enrollees with ADRD had a preventable hospitalization rate of 10.8% (95% CI, 10.3%-11.4%) in D-SNPs and 11.1% (95% CI, 10.7%-11.5%) in non–D-SNP MA plans (Table 2). There were no statistically significant differences between preventable hospitalization rates by D-SNP enrollment. Full regression results are available in eTable 3 in Supplement 1.

**Table 2.** Differences in Adjusted Rates of Adverse Outcomes by Enrollment Type, Among Dual-Eligible Medicare Beneficiaries With ADRD, 2017-2019^a^

Outcome	Rate, % (95% CI)		Difference, pp (95% CI)^b^
	Non–D-SNP MA	D-SNP	
Adjusted preventable hospitalization^c^	11.1 (10.7 to 11.5)	10.8 (10.3 to 11.4)	−0.3 (−0.7 to 0.2)
Adjusted all-cause 30-d readmission rates^c^	23.6 (22.9 to 24.3)	22.3 (21.6 to 22.9)	−1.3 (−2.1 to −0.6)
Adjusted avoidable ED visit rates^c^	20.8 (20.1 to 21.6)	21.1 (20.3 to 21.9)	0.3 (−0.2 to 0.8)

Abbreviations: D-SNP, dual-eligible special need plans; ED, emergency department.

^a^ Regression analyses controlled for age, sex, race and ethnicity, individual Centers for Medicare and Medicaid Services Hierarchical Condition Category score, frailty, median zip code–level income and education levels, rurality, and county-level care resources including hospital beds and physicians per 1000 population. Full regression results are presented in eAppendix 4 in Supplement 1.

^b^ Average marginal effects were calculated after regressions, as the percentage-point (pp) differences of adverse event probabilities in non–D-SNP MA enrollees vs D-SNP enrollees.

^c^ Adjusted rates were calculated after regressions, as the average estimated probabilities of having an outcome equaling 1 within each enrollment type.

Dual-eligible enrollees with ADRD had high rates of 30-day readmissions: 22.3% (95% CI, 21.6%-22.9%) in D-SNPs and 23.6% (95% CI, 22.9%-24.3%) in non–D-SNP MA plans. The probabilities of 30-day readmissions in D-SNP plans were 1.3 percentage points (95% CI, −2.1 to −0.6 percentage points) lower compared with those in non–D-SNP MA plans and the difference was statistically significant.

Avoidable ED visit rates did not differ by plan type. Dual-eligible enrollees with ADRD had avoidable ED use for 21.1% of those with D-SNP plans (95% CI, 20.3%-21.9%) and 20.8% of those with non–D-SNP MA plans (95% CI, 20.1%-21.6%).

Among enrollees with the highest projected risk, preventable hospitalization rates were 21% in both non–D-SNP and D-SNPs (a difference of 0.1 percentage points [95% CI, −0.7 to 0.8 percentage points]) (Figure). We observed 30% and 29% rates of 30-day readmissions, respectively, among dual-eligible beneficiaries with ADRD (a difference of −1.0 percentage points [95% CI, −2.1 to 0.1 percentage points]). The avoidable ED visit rates were 33% and 34% in non–D-SNP and D-SNP groups, respectively (a difference of 1.0 percentage points [95% CI, 0.1 to 2.0 percentage points]). The adjusted rates and adjusted differences between plan types are reported in eTable 4 in Supplement 1.

**Figure.**
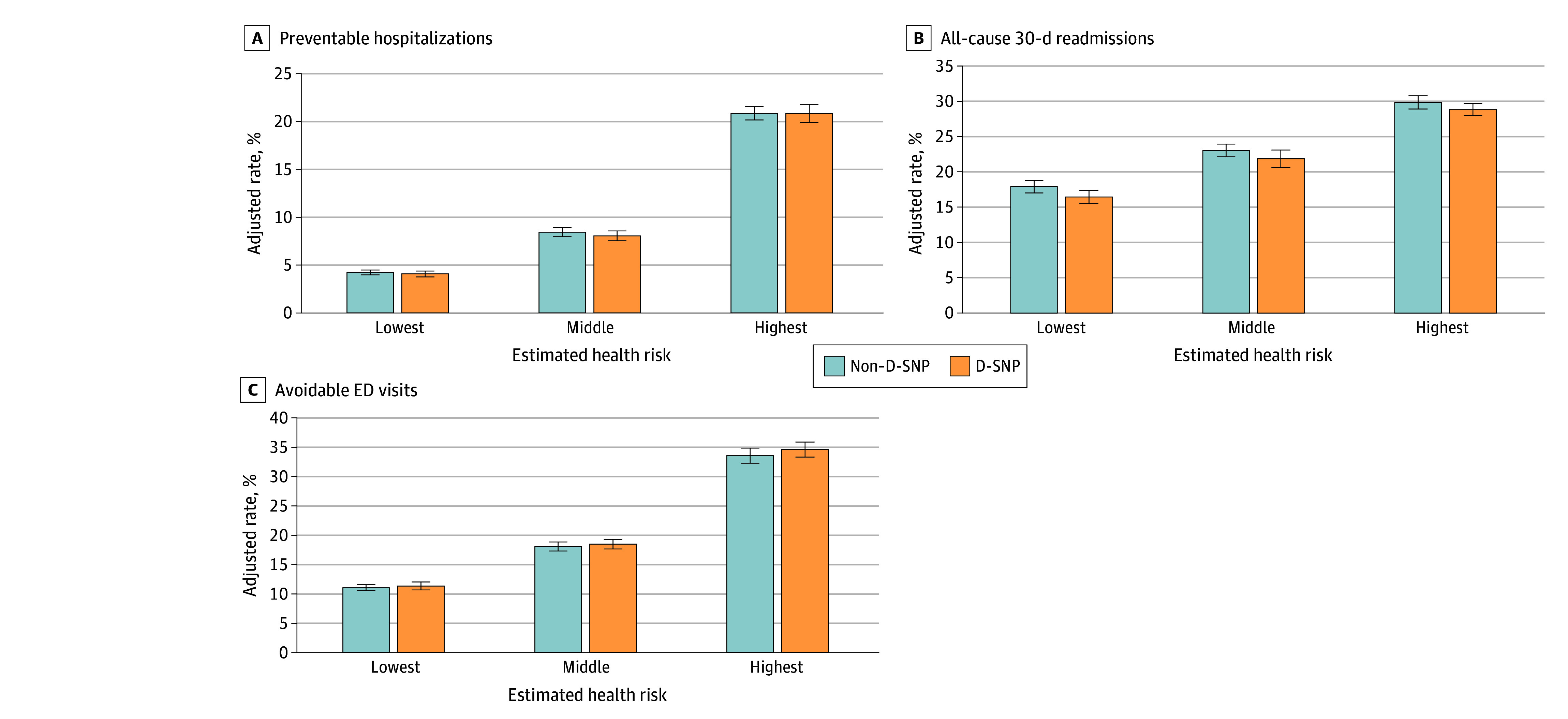
Adjusted Rates of Adverse Outcomes by Level of Projected Beneficiary Health Risk D-SNP indicates dual-eligible special needs plans; ED, emergency department.

The analyses expanded to FIDE SNPs found that compared with non–D-SNPs, enrollees in FIDE SNPs had lower probabilities of preventable hospitalizations by 1.2 percentage points (95% CI, −2.7 to −0.2 percentage points) and 30-day readmissions by 7.2 percentage points (95% CI, −9.3 to −5.1 percentage points) (Table 3). The difference in readmission rates between FIDE plans and D-SNP plans was also statistically significant. However, avoidable ED rates did not statistically differ by plan types (eTable 5 in Supplement 1).

**Table 3.** Differences in Adjusted Rates of Adverse Outcomes by Enrollment Type, Among Dual-Eligible Medicare Beneficiaries With ADRD, in States Where FIDE Plans Operated

Outcome	Non–D-SNP MA, % (95% CI)	D-SNP, % (95% CI)	D-SNP vs non–D-SNP, pp (95% CI)	FIDE, % (95% CI)	FIDE vs non–D-SNP, pp (95% CI)
Adjusted preventable hospitalization rates (95% CI)	10.3 (9.3 to 11.3)	10.1 (8.9 to 11.3)	−0.2 (−0.9 to 0.5)	9.0 (8.3 to 9.8)	−1.2 (−2.7 to −0.2)^a^
Adjusted all-cause 30-d readmission rates (95% CI)	23.9 (22.5 to 25.4)	22.5 (20.8 to 24.2)	−1.4 (−3.0 to 0.2)	16.8 (14.2 to 19.3)	−7.2 (−9.3 to −5.1)^a^^,^^b^
Adjusted avoidable ED visit rates (95% CI)	19.7 (17.9 to 21.6)	20.2 (18.0 to 22.4)	0.4 (−0.8 to 1.7)	18.4 (15.5 to 21.4)	−1.3 (−3.0 to 0.3)

Abbreviations: D-SNP, dual-eligible special need plans; ED, emergency department; FIDE, fully integrated dual-eligible; MA, Medicare Advantage; pp, percentage point.

^a^ Indicates statistical differences at 95% CI between non–D-SNP MA enrollees and D-SNP enrollees, and between non–D-SNP MA enrollees and FIDE plan enrollees.

^b^ Indicates statistical differences at 95% CI between D-SNP enrollees and FIDE plan enrollees.

The results from our sensitivity analysis using dual-eligible beneficiaries with full Medicaid benefits were similar to those of the primary analyses (eTable 6 in Supplement 1). Likewise, considering ED visits due to behavioral diagnoses did not change the results from the primary analysis (eTable 7 in Supplement 1). Findings from a probit model were similar to the main analysis (eTable 8 in Supplement 1). Sensitivity analysis with data for each year—2017, 2018, and 2019—produced consistent findings (eTable 9 in Supplement 1).

## Discussion

Medicare Advantage plans continue to experience prodigious growth with over half of the Medicare population enrolled in MA plans in 2024. MA plans that aim to better coordinate care and benefits for dual-eligible beneficiaries, such as D-SNPs, are experiencing particularly rapid growth. In this national study of dual-eligible beneficiaries with ADRD, we found that in general, coordination-only D-SNPs did not perform better than non–D-SNP MA plans in reducing adverse outcomes among dual-eligible beneficiaries with ADRD, except for a slightly lower 30-day readmission rate. Enrollees in D-SNPs and non–D-SNP MA plans experienced similar rates of preventable hospitalizations and avoidable ED visits. D-SNPs are intended to enhance care for dual-eligible beneficiaries, which can particularly help those with more intensive care needs and greater frailty. However, for dual-eligible beneficiaries with ADRD, enrollment in D-SNPs did not make a significant difference in the rates of adverse outcomes from non–D-SNPs among patients with higher health risks.

Our findings that D-SNPs offered little advantage over non–D-SNP MA plans in improving health of dual-eligible beneficiaries with ADRD are consistent with a growing body of evidence that found dual-eligibles did not experience better care in D-SNPs.^8,9,10^ However, enrollees in FIDE SNPs had lower rates of preventable hospitalizations and significantly lower rates of 30-day readmissions than those in non–D-SNP MA plans. They also had a lower rate of 30-day readmissions compared with enrollees in D-SNPs. These findings suggest that CMS may help improve care for frail dual-eligible beneficiaries by promoting more integrated plan models such as FIDE SNPs.

Dual-eligible beneficiaries with ADRD may frequently experience barriers to obtaining care due to a lack of resources and cognitive impairments. Coordination-only D-SNPs only offer minimal integration support to remove challenges to navigating the bifurcated system of Medicare and Medicaid. With more fully integrated design and coverage from one managed care organization, FIDE SNPs may help enhance continuous and sufficient ambulatory care, compared with coordination-only D-SNPs. Yet the small difference in preventable hospitalization and lack of difference in avoidable ED rates by FIDE SNP enrollment may reflect the fact that some FIDE SNPs did not fully align Medicare and Medicaid as designed, reducing the opportunity to improve patient outcomes.^34^

As an important national goal, HealthyPeople 2030 has prioritized reducing preventable hospitalizations for those with ADRD.^35^ Our findings suggest that the policy goals of reducing avoidable hospitalizations and readmissions in beneficiaries with ADRD are more likely to be successful with models of care that enable greater integration of Medicare and Medicaid benefits to enhance care coordination.

### Limitations

Several limitations of this study are worth mentioning. First, the study does not produce causal inferences. Even with IPTW, unobserved factors may still exist that bias the estimated outcomes. Second, the findings may not generalize to patients with ADRD who were not dual-eligible or those who were not community-dwelling. Furthermore, we did not have access to data from more recent years when FIDE SNPs expanded to more states, although enrollment in FIDE SNPs remained less than 5% in 2024. Moreover, it was beyond the scope of this study to characterize differences in utilization within D-SNPs or within other MA plans for beneficiaries with ADRD. Differences in plans’ care management strategies, provider networks, and level of Medicaid integration could affect the degree to which D-SNPs influence utilization. Finally, our findings may not generalize to beneficiaries without dementia.

## Conclusions

Enrollment in coordination-only D-SNPs was not associated with lower probabilities of preventable hospitalizations or avoidable ED visits than in non–D-SNP MA plans. Enrollment in D-SNP was associated with a small reduction in 30-day readmissions. However, enrollment in FIDE SNPs led to fewer preventable hospitalizations and readmissions. Dual-eligible beneficiaries with ADRD may benefit from enrollment in more coordinated plans by avoiding unnecessary hospitalizations.

## Notes

Supplement 1. **eAppendix.** Inclusion and Exclusion Criteria for Study Population **eTable 1.** Propensity Score Weighted Sample Characteristics of Dually Eligible Patients with ADRD, by Enrollment Type, 2017-2019 **eFigure 1.** Love Plots of Covariate Balance Before and After Inverse Probability of Treatment Weighting **eFigure 2.** Distribution of Propensity Scores for Non-D-SNP MA and D-SNP Enrollment, by Observed Enrollment Group **eTable 2.** Sample Characteristics, at Admission-Level, for Patients With ADRD, 2017-2019 **eTable 3.** Regression Estimates for Outcome Measures **eTable 4.** Differences in Adjusted Rates of Outcome Measures by Enrollment Type, Among Dual-Eligible Medicare Beneficiaries with ADRD, by Predicted Health Risks **eTable 5.** Adjusted Regression Estimates for Outcome Measures Among Dual-Eligible Enrollees with ADRD in States With FIDE Plans Available **eTable 6.** Adjusted Regression Estimates for Outcome Measures Among Dual-Eligible Enrollees with ADRD Who Had Full Medicaid Benefits **eTable 7.** Adjusted Regression Estimates for Outcomes Considering ED Visits for Psychiatric Conditions **eTable 8.** Probit Regression Estimates for Outcome Measures **eTable 9.** Differences in Adjusted Rates of Outcome Measures by Enrollment Type, Among Dual-Eligible Medicare Beneficiaries With ADRD, by Year

Supplement 2.
Data Sharing Statement
