# Supplementary material for: Outcomes for Dual-Eligible Beneficiaries With Dementia in Special Needs Plans and Other Medicare Advantage Plans
Source: JAMA Netw Open. 2025 Feb 21;8(2):e2461219. doi: 10.1001/jamanetworkopen.2024.61219 (PMC11846004; doi:10.1001/jamanetworkopen.2024.61219)
Supplement: Supplement 1. — eAppendix. Inclusion and Exclusion Criteria for Study Population eTable 1. Propensity Score Weighted Sample Characteristics of Dually Eligible Patients with ADRD, by Enrollment Type, 2017-2019 eFigure 1. Love Plots of Covariate Balance Before and After Inverse Probability of Treatment Weighting eFigure 2. Distribution of Propensity Scores for Non-D-SNP MA and D-SNP Enrollment, by Observed Enrollment Group eTable 2. Sample Characteristics, at Admission-Level, for Patients With ADRD, 2017-2019 eTable 3. Regression Estimates for Outcome Measures eTable 4. Differences in Adjusted Rates of Outcome Measures by Enrollment Type, Among Dual-Eligible Medicare Beneficiaries with ADRD, by Predicted Health Risks eTable 5. Adjusted Regression Estimates for Outcome Measures Among Dual-Eligible Enrollees with ADRD in States With FIDE Plans Available eTable 6. Adjusted Regression Estimates for Outcome Measures Among Dual-Eligible Enrollees with ADRD Who Had Full Medicaid Benefits eTable 7. Adjusted Regression Estimates for Outcomes Considering ED Visits for Psychiatric Conditions eTable 8. Probit Regression Estimates for Outcome Measures eTable 9. Differences in Adjusted Rates of Outcome Measures by Enrollment Type, Among Dual-Eligible Medicare Beneficiaries With ADRD, by Year [file jamanetwopen-e2461219-s001.pdf]

Supplemental Online Content

Xu W, Raver E, Carlin C, Feldman R, Retchin SM, Jung J. Outcomes of dual-eligibles with dementia in special needs plans and other Medicare Advantage plans. *JAMA Netw Open.* 2025;8(2):e2461219. doi:10.1001/jamanetworkopen.2024.61219

eAppendix. Inclusion and Exclusion Criteria for Study Population

eTable 1. Propensity Score Weighted Sample Characteristics of Dually Eligible Patients with ADRD, by Enrollment Type, 2017-2019

eFigure 1. Love Plots of Covariate Balance Before and After Inverse Probability of Treatment Weighting

eFigure 2. Distribution of Propensity Scores for Non-D-SNP MA and D-SNP Enrollment, by Observed Enrollment Group

eTable 2. Sample Characteristics, at Admission-Level, for Patients With ADRD, 2017-2019

eTable 3. Regression Estimates for Outcome Measures

eTable 4. Differences in Adjusted Rates of Outcome Measures by Enrollment Type, Among Dual-Eligible Medicare Beneficiaries with ADRD, by Predicted Health Risks

eTable 5. Adjusted Regression Estimates for Outcome Measures Among Dual-Eligible Enrollees with ADRD in States With FIDE Plans Available

eTable 6. Adjusted Regression Estimates for Outcome Measures Among Dual-Eligible Enrollees with ADRD Who Had Full Medicaid Benefits

eTable 7. Adjusted Regression Estimates for Outcomes Considering ED Visits for Psychiatric Conditions

eTable 8. Probit Regression Estimates for Outcome Measures

eTable 9. Differences in Adjusted Rates of Outcome Measures by Enrollment Type, Among Dual-Eligible Medicare Beneficiaries With ADRD, by Year

This supplemental material has been provided by the authors to give readers additional information about their work.

© 2025 Xu W et al. *JAMA Network Open*.

eAppendix. Inclusion and Exclusion Criteria for Study Population

The study population comprised dual-eligible, community-dwelling Medicare Advantage (MA) beneficiaries aged 65 and older with ADRD. Since D-SNPs are tailored to support care infrastructure for community dwellers,[21] long-stay nursing home residents were not in the study (37% of dual-eligible enrollees 65 and above with ADRD). We used the Minimum Data Set nursing home assessment data to identify long-stay patients based on the CMS definition of “long stay” as >100 days in a nursing home.[22 23] Medicare beneficiaries who enrolled in either full or partial Medicaid programs any month of a year were considered dual eligibles. Following the algorithm the Centers for Medicare and Medicaid Services (CMS) uses for the Medicare population, patients with ADRD were identified by having at least one inpatient claim or two outpatient claims with ADRD diagnosis codes in a two-year reference window.[16] The inclusion criteria further required community-dwelling beneficiaries to reside in 50 states or Washington D.C., with full Medicare Part A, B, D coverage. We included only enrollees in MA contracts with highly complete encounter data, following a validated method in the literature (this dropped about 17% of the population).[19 20]

We excluded beneficiaries who switched between MA plans during a year (4.5% of the population). Because of the different plan structures and provider network requirements, we excluded those in Program of All-Inclusive Care for the Elderly plans or Cost Plans, or in private fee-for-service plans. Beneficiaries were also excluded if they lived outside the 50 states or Washington D.C. or if their current Medicare eligibility was due to end-stage renal disease. These criteria excluded about 0.9% of the population. We also excluded enrollees in SNPs designed mainly for institutional care and for those with chronic health conditions (3.4% of the population).

© 2025 Xu W et al. *JAMA Network Open*.

eTable 1. Propensity Score Weighted Sample Characteristics of Dually Eligible Patients with ADRD, by Enrollment Type, 2017-2019

	Person-Year Sample		
Characteristic	Non-D-SNP Medicare Advantage	Dual Eligible Special Needs Plans	Standardized Mean Difference*
Age, years	81.0	81.0	-0.006
Female, %	70.7	70.6	-0.001
Race and ethnicity, %:	--	--	--
White, non-Hispanic	44.0	43.3	-0.007
Black, non-Hispanic	23.3	23.6	0.004
Asian or Pacific Islander	5.9	5.6	-0.007
Hispanic	26.9	27.5	0.009
Rural residence, %	13.7	13.6	-0.001
HCC risk score	2.1	2.2	-0.008
Kim frailty score	0.22	0.22	-0.007
Median household income (ZIP code level), $	55142	55362	0.004
Percentage of ZIP code with a 4-year degree	25.8	25.8	0.000
Percentage of ZIP code households speaking only English	70.9	70.3	-0.015
Percentage of ZIP code households under the Federal Poverty Level §	17.7	17.7	0.001
Hospital beds per 1000 population (county level)	3.2	3.2	-0.002
Skilled nursing facility beds per 1000 population (county level) §	5.1	5.0	-0.014
Physicians per 1000 population (county level)	3.7	3.7	-0.001
Primary care providers per 1000 population (county level) §	0.7	0.7	0.002
Medicare Advantage county HHI	2081	2071	-0.005
Medicare Advantage county penetration, % §	43.8	43.9	0.004

© 2025 Xu W et al. *JAMA Network Open*.

§ Percentage of ZIP code households under the Federal Poverty Level, skilled nursing facility beds per 1000 population (county level), primary care providers per 1000 population (county level), and Medicare Advantage County penetration were only included in propensity weighting, but not in main regression analyses.

* The standardized mean difference represents the standard deviation difference in a characteristic between the two enrollment groups.

© 2025 Xu W et al. *JAMA Network Open*.

eFigure 1. Love plots of covariate balance before and after inverse probability of treatment weighting

The Love plot displays the standardized mean differences in covariates between non-D-SNP MA enrollees and D-SNP enrollees, before and after implementing inverse probability of treatment weighting (IPTW). For example, the average age of D-SNP enrollees before IPTW was 0.21 standard deviations less than that of other MA enrollees. After IPTW, the average age of D-SNP enrollees was 0.006 standard deviations less than that of other MA enrollees. A standardized mean difference between -0.1 and 0.1 indicates well balanced covariates between the two groups. The Love plots show that the IPTW worked to balance the non-D-SNP MA and D-SNP groups for both the person-year sample and admission-level sample.

Panel A. Person-year sample

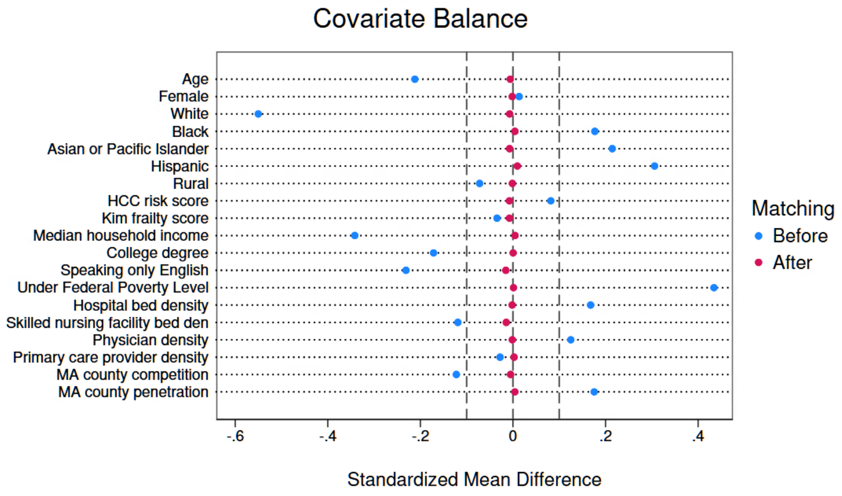

Panel B. Admission-level sample

© 2025 Xu W et al. *JAMA Network Open*.

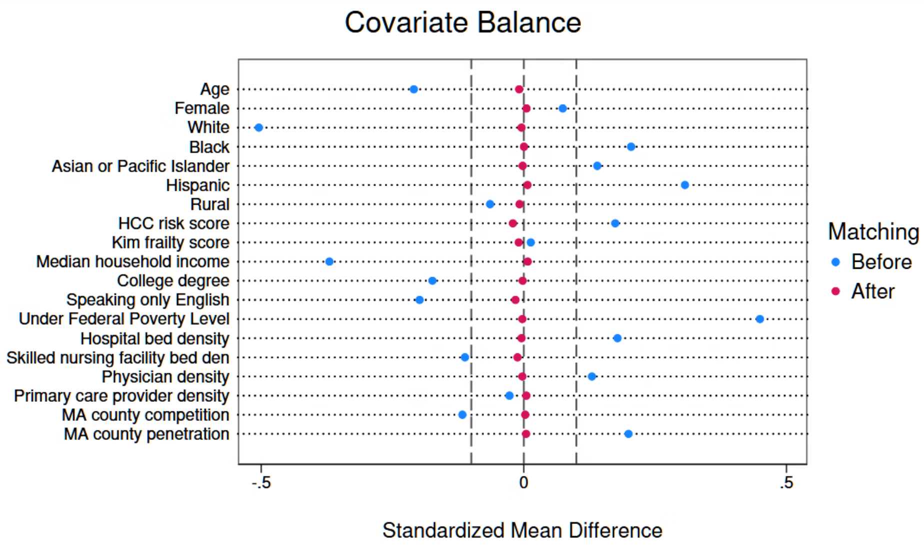

© 2025 Xu W et al. *JAMA Network Open*.

eFigure 2. Distribution of propensity scores for non-D-SNP MA and D-SNP enrollment, by observed enrollment group

The x-axis indicates the propensity score for D-SNP enrollment, which is the *predicted* likelihood of being enrolled in a D-SNP. This is displayed by *observed* enrollment group, with non-D-SNP MA enrollees represented by the solid blue line, and D-SNP enrollees represented by the dashed red line. The propensity score was calculated from logistic regression analysis that controlled for age, sex, race and ethnicity, median ZIP code-level income, ZIP-code level poverty and education levels, rurality, county-level care resources including hospital beds, skilled nursing facility beds, physicians, and primary care providers per 1000 population, and Medicare Advantage penetration in a county. The y-axis indicates probability density, which is the relative likelihood of having a particular propensity score, for those in a given enrollment group.

The propensity score distributions for the non-D-SNP MA and D-SNP groups showed that there were valid comparisons between groups over a wide range of likelihoods for being enrolled in one group or another. We excluded observations outside the area of common support by the blue and red lines, where individuals have a non-zero likelihood of being enrolled in either non-D-SNP MA or D-SNP.

Panel A. Person-year sample

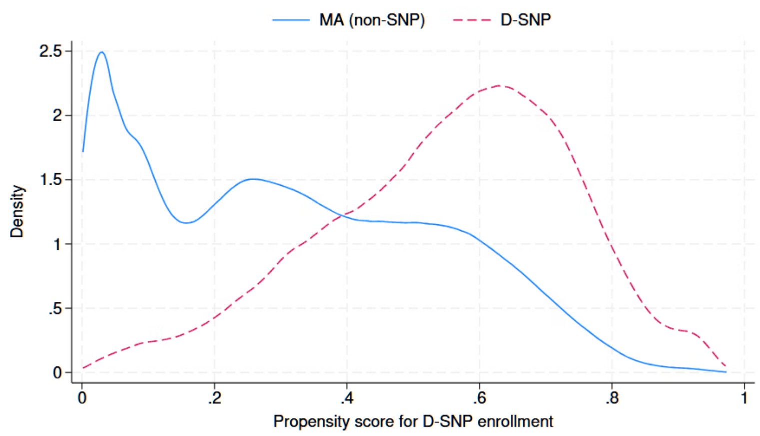

Panel B. Admission-level sample

© 2025 Xu W et al. *JAMA Network Open*.

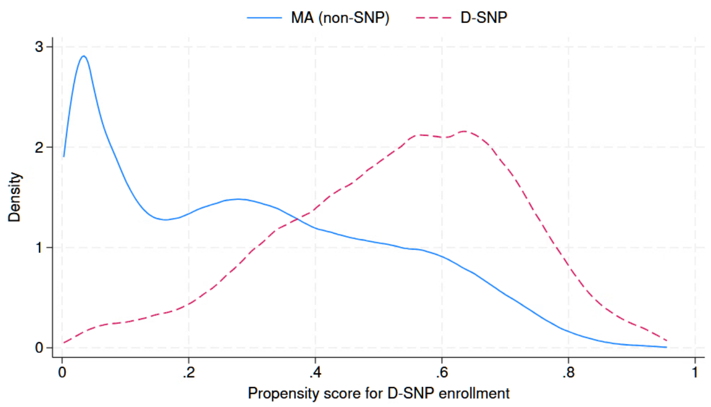

© 2025 Xu W et al. *JAMA Network Open*.

eTable 2. Sample Characteristics, at Admission-Level, for Patients with ADRD, 2017-2019

**Characteristics**	**Admission-Level Sample: 30-Day All-Cause Readmissions**	
	**Non-DSNP Medicare Advantage Plans**	**D-SNPs**
Number of Admission Observations	123,288	78,341
Number of Patients	60,737	36,893
Number of Health Plans	1916	289
Age, mean (SD)	80.8 (7.9)	79.1 (8.0)
Female, n (%)	80,572 (65.4)	53,924 (68.9)
White (non-Hispanic), n (%)	72,481 (58.8)	26,945 (34.4)
Black (non-Hispanic), n (%)	27,004 (21.9)	24,174 (30.9)
Asian (non-Hispanic), n (%)	2832 (2.3)	3834 (4.9)
Hispanic (any race), n (%)	20,971 (17.0)	23,388 (29.9)
Rural, n (%)	18,477 (15.0)	10,002 (12.8)
HCC score, mean (SD)	2.7 (2.0)	3.1 (2.0)
Frailty score, mean (SD)	0.28 (0.10)	0.28 (0.10)
Median household income (ZIP code level), mean (SD)	57863 (22540)	50026 (19715)
Percentage of ZIP code with a 4-year degree, mean (SD)	26.6 (14.5)	24.1 (14.1)
Percentage of ZIP code households speaking only English, mean (SD)	75.7 (24.3)	70.7 (26.4)
Hospital beds per 1000 population (county level), mean (SD)	3.1 (2.0)	3.5 (2.0)
Physicians per 1000 population (county level), mean (SD)	3.6 (2.3)	3.9 (2.8)
Medicare Advantage county HHI, mean (SD)	2132 (1021)	2014 (1000)

© 2025 Xu W et al. *JAMA Network Open*.

eTable 3. Regression Estimates for Outcome Measures

Percentage point difference (95% CI)

	**Preventable Hospitalizations**	**30-day readmissions**	**Avoidable ED Visits**
**Enrollment (ref: non-D-SNP MA plans)**	--	--	--
**D-SNP**	-0.3 (-0.7, 0.2)	-1.3 (-2.1, -0.6)	0.3 (-0.2, 0.8)
Age, years	0.0 (-0.1, 0.0)	-0.5 (-0.5, -0.4)	-0.2 (-0.2, -0.2)
Female (ref: Male)	0.3 (-0.1, 0.8)	-1.9 (-2.5, -1.2)	-0.5 (-1.1, -0.0)
Race/ethnicity (ref: White (non-Hispanic)	--	--	--
Black (non-Hispanic)	-0.3 (-0.8, 0.3)	0.1 (-0.8, 1.0)	0.1 (-0.6, 0.8)
Asian (non-Hispanic)	-1.9 (-3.0, -0.7)	-1.2 (-2.9, 0.6)	-5.9 (-7.3, -4.4)
Hispanic (any race)	-1.6 (-2.3, -1.0)	-1.8 (-2.9, -0.7)	-1.7 (-2.5, -0.9)
Rural (ref: Urban)	-1.2 (-3.0, 0.7)	0.1 (-1.6, 1.8)	-0.4 (-2.4, 1.7)
HCC score	2.7 (2.6, 2.9)	1.5 (1.3, 1.7)	2.7 (2.5, 2.9)
Frailty score	70.1 (66.8, 73.3)	38.1 (34.3, 41.9)	100.3 (95.8. 104.9)
Median household income (ZIP code level), $ (SD)	0.0 (0.0, 0.0)	0.0 (0.0, 0.0)	0.0 (0.0, 0.0)
Percentage of ZIP code with a 4-year degree, %	0.0 (0.0, 0.0)	0.0 (0.0, 0.1)	0.0 (0.0, 0.0)
Percentage of ZIP code households speaking only English, %	0.0 (0.0, 0.0)	0.0 (-0.1, 0.0)	0.0 (0.0, 0.0)
Hospital beds per 1000 population (county level)	0.1 (-0.1, 0.3)	-0.1 (-0.5, 0.4)	0.3 (-0.1, 0.8)
Physicians per 1000 population (county level)	-0.3 (-0.5, -0.1)	0.3 (-0.3, 0.8)	-0.6 (-0.9, -0.2)
Medicare Advantage county HHI	0.0 (0.0, 0.0)	0.0 (0.0, 0.0)	0.0 (0.0, 0.0)
Number of Patients	199,311	97,630	199,311
Number of Patient-Year or Admission Observations	296,343	201,629	296,343

Note:
§ Regression models control for county and year fixed effects.

© 2025 Xu W et al. *JAMA Network Open*.

eTable 4. Differences in Adjusted Rates of Outcome Measures by Enrollment Type, Among Dual-Eligible Medicare Beneficiaries with ADRD, by Predicted Health Risks

Preventable Hospitalizations		
	**non-D-SNP MA Plans**	**D-SNP**
**Percentage-point differences compared to non-D-SNP MA Plans**, Lowest Health Risk Tercile (95% CI)	--	-0.2 (-0.5, 0.2)
Adjusted Rates, Lowest Health Risk Tercile [95% CI]	4.2 [4.0, 4.5]	4.1 [3.8, 4.4]
**Percentage-point differences compared to non-D-SNP MA Plans**, Middle Health Risk Tercile (95% CI)	--	-0.4 (-0.8, 0.0)
Adjusted Rates, Middle Health Risk Tercile [95% CI]	8.4 [7.9, 8.9]	8.0 [7.5, 8.5]
**Percentage-point differences compared to non-D-SNP MA Plans**, Highest Health Risk Tercile (95% CI)	--	0.1 (-0.7, 0.8)
Adjusted Rates, Highest Health Risk Tercile [95% CI]	20.7 [19.9, 21.5]	20.8 [19.8, 21.7]
**All-Cause 30-day Readmissions**		
	**Non-D-SNP MA Plans**	**D-SNP**
**Percentage-point differences compared to non-D-SNP MA Plans**, Lowest Health Risk Tercile (95% CI)	--	-1.5 (-2.5, -0.5)
Adjusted Rates, Lowest Health Risk Tercile [95% CI]	17.9 [17.0, 18.8]	16.4 [15.5, 17.4]
**Percentage-point differences compared to non-D-SNP MA Plans**, Middle Health Risk Tercile (95% CI)	--	-1.2 (-2.4, -0.1)
Adjusted Rates, Middle Health Risk Tercile [95% CI]	23.0 [22.1, 23.9]	21.8 [20.6, 23.1]
**Percentage-point differences compared to non-D-SNP MA Plans**, Highest Health Risk Tercile (95% CI)	--	-1.0 (-2.1, 0.1)

© 2025 Xu W et al. *JAMA Network Open*.

Adjusted Rates, Highest Health Risk Tercile<br>[95% CI]	29.8<br>[28.9, 30.8]	28.8<br>[28.0, 29.7]
**Avoidable ED Visits**		
	**Non-D-SNP MA Plans**	**D-SNP**
**Percentage-point differences compared to non-D-SNP MA Plans**, Lowest Health Risk Tercile<br>(95% CI)	--	0.3<br>(-0.3, 0.9)
Adjusted Rates, Lowest Health Risk Tercile<br>[95% CI]	11.0<br>[10.5, 11.5]	11.3<br>[10.6, 11.9]
**Percentage-point differences compared to non-D-SNP MA Plans**, Middle Health Risk Tercile<br>(95% CI)	--	0.4<br>(-0.2, 1.0)
Adjusted Rates, Middle Health Risk Tercile<br>[95% CI]	18.0<br>[17.2, 18.7]	18.3<br>[17.5, 19.2]
**Percentage-point differences compared to non-D-SNP MA Plans**, Highest Health Risk Tercile<br>(95% CI)	--	1.0<br>(0.1, 2.0)
Adjusted Rates, Highest Health Risk Tercile<br>[95% CI]	33.3<br>[32.1, 34.6]	34.4<br>[33.1, 35.6]

-Health risk terciles were predicted for a 90% testing sample based on regressions of the adverse event on HCC risk score and frailty score in a separate 10% training sample. We chose a 10% training sample based on simulations that showed consistent health risk predictions for models based on training samples ranging from 10% to 80%. Regression analyses controlled for age, sex, race/ethnicity, median ZIP code-level income and education levels, rurality, and county-level care resources including hospital beds and physicians per 1000 population.

-Adjusted rates were calculated after regressions, as the average predicted probabilities of having an outcome=1, within each enrollment type.

-Percentage-point differences: Averaged marginal effects were calculated after regressions, as the percentage-point differences of adverse event probabilities in D-SNP enrollees vs. non-D-SNP MA enrollees.

© 2025 Xu W et al. *JAMA Network Open*.

eTable 5. Adjusted Regression Estimates for Outcome Measures Among Dual-Eligible Enrollees with ADRD in States With FIDE Plans Available
Percentage point difference (95% CI)

	**Preventable Hospitalizations**	**30-day readmissions**	**Avoidable ED Visits**
**Enrollment (ref: non-D-SNP Medicare Advantage)**	--	--	--
**D-SNP**	-0.2 (-0.9, 0.5)	-1.4 (-3.0, 0.2)	0.4 (-0.8, 1.7)
**FIDE**	-1.2 (-2.7, -0.2)	-7.2 (-9.3, -5.1)	-1.3 (-3.0, 0.3)
Age, years	0.1 (0.0, 0.1)	-0.5 (-0.6, -0.4)	-0.3 (-0.4, -0.1)
Female (ref: Male)	0.9 (-0.3, 2.1)	-2.7 (-5.1, -0.3)	1.1 (-2.4, 4.6)
Race/ethnicity (ref: White (non-Hispanic)	--	--	--
Black (non-Hispanic)	2.3 (-1.9, 6.6)	0.0 (-1.5, 1.5)	-1.5 (-4.0, 0.9)
Asian (non-Hispanic)	-3.0 (-4.4, -1.5)	-1.6 (-4.0, 0.8)	-6.8 (-9.5, -4.0)
Hispanic (any race)	-0.2 (-2.9, 2.4)	2.1 (-2.8, 7.0)	0.2 (-1.1, 1.6)
Rural (ref: Urban)	0.7 (-2.1, 3.6)	-1.9 (-6.0, 2.2)	-1.7 (-5.6, 2.2)
HCC score	2.7 (2.3, 3.1)	1.7 (1.4, 2.0)	2.4 (2.0, 2.8)
Frailty score	52.0 (35.7, 68.3)	34.5 (27.5, 41.5)	87.0 (77.1, 96.8)
Median household income (ZIP code level), $ (SD)	0.0 (0.0, 0.0)	0.0 (0.0, 0.0)	0.0 (0.0, 0.0)
Percentage of ZIP code with a 4-year degree, %	0.0 (-0.1, 0.1)	0.0 (0.0, 0.1)	0.1 (-0.1, 0.3)
Percentage of ZIP code households speaking only English, %	0.0 (-0.1, 0.0)	0.0 (0.0, 0.1)	0.0 (0.0, 0.0)
Hospital beds per 1000 population (county level)	-0.2 (-1.1, 0.6)	-1.0 (-1.9, 0.0)	-0.7 (-2.8, 1.3)
Physicians per 1000 population (county level)	0.2 (-0.4, 0.8)	0.5 (-0.1, 1.1)	-0.2 (-1.2, 0.9)
Medicare Advantage county HHI	0.0 (0.0, 0.0)	0.0 (0.0, 0.0)	0.0 (0.0, 0.0)
Number of Patients	94,381	42,519	94,381
Number of Patient-Year or Admission Observations	131,417	83,769	131,417

Note:
§ Regression models control for county and year fixed effects.

© 2025 Xu W et al. *JAMA Network Open*.

eTable 6. Adjusted Regression Estimates for Outcome Measures Among Dual-Eligible Enrollees with ADRD Who Had Full Medicaid Benefits

Percentage point difference (95% CI)

	**Preventable Hospitalizations**	**30-day readmissions**	**Avoidable ED Visits**
**Enrollment (ref: non-D-SNP MA plans)**	--	--	--
**D-SNP**	-0.5 (-1.0, -0.1)	-1.9 (-2.9, -0.9)	-0.1 (-0.7, 0.5)
Age, years	0.0 (-0.1, 0.0)	-0.5 (-0.5, -0.4)	-0.2 (-0.3, -0.2)
Female (ref: Male)	0.3 (-0.2, 0.8)	-1.8 (-2.6, -0.9)	-0.2 (-0.9, 0.4)
Race/ethnicity (ref: White (non-Hispanic)	--	--	--
Black (non-Hispanic)	0.1 (-0.6, 0.8)	0.2 (-1.1, 1.6)	0.2 (-0.6, 1.1)
Asian (non-Hispanic)	-1.8 (-2.8, -0.7)	-1.1 (-3.2, 1.1)	-6.3 (-7.8, -4.7)
Hispanic (any race)	-1.3 (-2.0, -0.7)	-1.6 (-3.0, -0.1)	-1.4 (-2.3, -0.5)
Rural (ref: Urban)	-0.6 (-2.7, -1.5)	0.3 (-1.9, 2.4)	0.6 (-2.1, 3.2)
HCC score	2.7 (2.5, 2.9)	1.5 (1.3, 1.7)	2.6 (2.3, 2.8)
Frailty score	68.3 (64.8, 71.8)	37.0 (31.9, 42.1)	97.6 (92.2, 103.0)
Median household income (ZIP code level), $ (SD)	0.0 (0.0, 0.0)	0.0 (0.0, 0.0)	0.0 (0.0, 0.0)
Percentage of ZIP code with a 4-year degree, %	0.0 (-0.1, 0.0)	0.0 (0.0, 0.1)	0.0 (0.0, 0.0)
Percentage of ZIP code households speaking only English, %	0.0 (0.0, 0.0)	0.0 (0.1, 0.0)	0.0 (0.0, 0.0)
Hospital beds per 1000 population (county level)	0.2 (0.0, 0.5)	0.1 (-0.6, 0.7)	0.5 (0.0, 1.0)
Physicians per 1000 population (county level)	-0.4 (-0.6, -0.1)	0.2 (-0.5, 1.0)	-0.7 (-1.1, -0.2)
Medicare Advantage county HHI	0.0 (0.0, 0.0)	0.0 (0.0, 0.0)	0.0 (0.0, 0.0)
Number of Patients	145,126	73,089	145,126
Number of Patient-Year or Admission Observations	207,998	150,103	207,998

Note:
§ Regression models control for county and year fixed effects.

© 2025 Xu W et al. *JAMA Network Open*.

eTable 7. Adjusted Regression Estimates for Outcomes Considering ED Visits for Psychiatric Conditions

Percentage point difference (95% CI)

	**Any ED Visit**	**Any ED Visit for Psychiatric Conditions**	**Avoidable ED Visit or ED Visit for Psychiatric Conditions**
**Enrollment (ref: non-D-SNP Medicare Advantage)**	--	--	--
**D-SNP**	-0.5 (-1.5, 0.4)	-0.4 (-0.6, -0.2)	0.0 (-0.5, 0.6)
Age, years	0.0 (0.0, 0.0)	-0.2 (-0.2, -0.1)	-0.3 (-0.3. -0.3)
Female (ref: Male)	-1.9 (-2.4, -1.3)	-0.6 (-0.8, -0.4)	-1.0 (-1.5, -0.4)
Race/ethnicity (ref: White (non-Hispanic)	--	--	--
Black (non-Hispanic)	0.8 (-0.2, 1.7)	-0.5 (-0.8, -0.2)	-0.1 (-0.8, 0.7)
Asian (non-Hispanic)	-15.7 (-18.0, -13.5)	-2.1 (-2.5, -1.6)	-7.3 (-8.8, -5.7)
Hispanic (any race)	-3.3 (-4.6, -2.0)	-0.9 (-1.2, -0.6)	-2.2 (-3.0, -1.4)
Rural (ref: Urban)	-3.3 (-6.8, 0.2)	0.0 (-0.8, 0.7)	-0.2 (-2.5, 2.1)
HCC score	2.0 (1.8, 2.2)	-0.3 (-0.4, -0.3)	2.3 (2.2, 2.5)
Frailty score	173.9 (170.4, 177.4)	16.0 (14.7, 17.3)	107.6 (103.0, 112.2)
Median household income (ZIP code level), $ (SD)	0.0 (0.0, 0.0)	0.0 (0.0, 0.0)	0.0 (0.0, 0.0)
Percentage of ZIP code with a 4-year degree, %	0.0 (-0.1, 0.0)	0.0 (0.0, 0.0)	0.0 (0.0, 0.0)
Percentage of ZIP code households speaking only English, %	0.0 (0.0, 0.1)	0.0 (0.0, 0.0)	0.0 (0.0, 0.0)
Hospital beds per 1000 population (county level)	0.0 (-0.5, 0.5)	0.0 (-0.1, 0.2)	0.4 (-0.1, 0.8)
Physicians per 1000 population (county level)	0.0 (-0.5, 0.5)	0.0 (-0.2, 0.1)	-0.5 (-0.9, -0.1)
Medicare Advantage county HHI	0.0 (0.0, 0.0)	0.0 (0.0, 0.0)	0.0 (0.0, 0.0)
Number of Patients	199,311	199,311	199,311
Number of Patient-Year or Admission Observations	296,343	296,343	296,343

Note:

§ Regression models control for county and year fixed effects.

© 2025 Xu W et al. *JAMA Network Open*.

eTable 8. Probit Regression Estimates for Outcome Measures
Percentage point difference (95% CI)

	**Preventable Hospitalizations**	**30-day readmissions**	**Avoidable ED Visits**
**Enrollment (ref: non-D-SNP MA plans)**	--	--	--
**D-SNP**	-0.3 (-0.8, 0.1)	-1.3 (-2.2, -0.4)	0.1 (-0.6, 0.7)
Age, years	0.0 (0.0, 0.0)	-0.5 (-0.5, -0.4)	-0.2 (-0.2, -0.2)
Female (ref: Male)	0.1 (-0.3, 0.6)	-2.2 (-2.9, -1.6)	-0.6 (-1.1, 0.0)
Race/ethnicity (ref: White (non-Hispanic)	--	--	--
Black (non-Hispanic)	-0.3 (-0.8, 0.2)	0.6 (-0.3, 1.4)	0.2 (-0.4, 0.8)
Asian (non-Hispanic)	-2.5 (-3.8, -1.3)	-0.7 (-2.6, 1.2)	-6.9 (-8.4, -5.3)
Hispanic (any race)	-2.0 (-2.7, -1.3)	-1.8 (-2.9, -0.8)	-2.3 (-3.1, -1.5)
Rural (ref: Urban)	-0.8 (-1.3, -0.3)	0.1 (-1.0, 1.2)	1.8 (1.0, 2.6)
HCC score	2.1 (1.9, 2.2)	1.3 (1.2, 1.5)	2.3 (2.1, 2.5)
Frailty score	59.6 (56.8, 62.5)	38.1 (34.5, 41.6)	91.8 (87.6, 96.0)
Median household income (ZIP code level), $ (SD)	0.0 (0.0, 0.0)	0.0 (0.0, 0.0)	0.0 (0.0, 0.0)
Percentage of ZIP code with a 4-year degree, %	0.0 (-0.1, 0.0)	0.0 (0.0, 0.0)	0.0 (-0.1, 0.0)
Percentage of ZIP code households speaking only English, %	0.0 (0.0, 0.0)	0.0 (0.0, 0.0)	0.1 (0.0, 0.1)
Hospital beds per 1000 population (county level)	0.0 (-0.1, 0.1)	0.1 (-0.1, 0.3)	0.2 (0.1, 0.4)
Physicians per 1000 population (county level)	0.0 (-0.1, 0.1)	0.1 (-0.1, 0.3)	-0.2 (-0.4, -0.1)
Medicare Advantage county HHI	0.0 (0.0, 0.0)	0.0 (0.0, 0.0)	0.0 (0.0, 0.0)
Number of Patients	199,311	97,630	199,311
Number of Patient-Year or Admission Observations	296,343	201,629	296,343

Note:
§ Regression models control for state and year fixed effects.

© 2025 Xu W et al. *JAMA Network Open*.

eTable 9. Differences in Adjusted Rates of Outcome Measures by Enrollment Type, Among Dual-Eligible Medicare Beneficiaries With ADRD, by Year

Preventable Hospitalizations		
	**non-D-SNP MA Plans**	**D-SNP**
**Percentage-point differences compared to non-D-SNP MA Plans**, 2017 (95% CI)	--	0.1 (-0.4, 0.7)
Adjusted Rates, 2017 [95% CI]	9.1 [8.6, 9.6]	9.2 [8.7, 9.7]
**Percentage-point differences compared to non-D-SNP MA Plans**, 2018 (95% CI)	--	-0.4 (-1.2, 0.4)
Adjusted Rates, 2018 [95% CI]	10.5 [9.9, 11.1]	10.2 [9.9, 11.2]
**Percentage-point differences compared to non-D-SNP MA Plans**, 2019 (95% CI)	--	-0.5 (-1.0, 0.0)
Adjusted Rates, 2019 [95% CI]	12.3 [11.8, 12.8]	11.8 [11.3, 12.4]
**All-Cause 30-day Readmissions**		
	**Non-D-SNP MA Plans**	**D-SNP**
**Percentage-point differences compared to non-D-SNP MA Plans**, 2017 (95% CI)	--	-1.1 (-2.2, -0.1)
Adjusted Rates, 2017 [95% CI]	19.9 [18.7, 21.0]	18.7 [17.6, 19.8]
**Percentage-point differences compared to non-D-SNP MA Plans**, 2018 (95% CI)	--	-1.9 (-2.8, -1.0)
Adjusted Rates, 2018 [95% CI]	21.4 [20.7, 22.1]	19.5 [18.7, 20.3]
**Percentage-point differences compared to non-D-SNP MA Plans**, 2019 (95% CI)	--	-1.3 (-2.5, -0.1)

© 2025 Xu W et al. *JAMA Network Open*.

Adjusted Rates, 2019<br>[95% CI]	26.2<br>[25.3, 27.1]	24.9<br>[23.9, 26.0]
**Avoidable ED Visits**		
	**Non-D-SNP MA Plans**	**D-SNP**
**Percentage-point differences compared to non-D-SNP MA Plans**, 2017<br>(95% CI)	--	0.9<br>(0.2, 1.6)
Adjusted Rates, 2017<br>[95% CI]	19.3<br>[18.3, 20.3]	20.2<br>[19.1, 21.2]
**Percentage-point differences compared to non-D-SNP MA Plans**, 2018<br>(95% CI)	--	-0.1<br>(-0.9, 0.6)
Adjusted Rates, 2018<br>[95% CI]	20.3<br>[19.4, 21.2]	20.2<br>[19.0, 21.3]
**Percentage-point differences compared to non-D-SNP MA Plans**, 2019<br>(95% CI)	--	0.1<br>(-0.5, 0.8)
Adjusted Rates, 2019<br>[95% CI]	21.8<br>[20.9, 22.7]	21.9<br>[21.1, 22.8]

- Regression analyses controlled for age, sex, race/ethnicity, median ZIP code-level income and education levels, rurality, and county-level care resources including hospital beds and physicians per 1000 population.

- Adjusted rates were calculated after regressions, as the average predicted probabilities of having an outcome=1, within each enrollment type.

- Percentage-point differences: Averaged marginal effects were calculated after regressions, as the percentage-point differences of adverse event probabilities in D-SNP enrollees vs. non-D-SNP MA enrollees.

© 2025 Xu W et al. *JAMA Network Open*.
